# Contribution to Speeding-Up the Solving of Nonlinear Ordinary Differential Equations on Parallel/Multi-Core Platforms for Sensing Systems

**DOI:** 10.3390/s20216130

**Published:** 2020-10-28

**Authors:** Vahid Tavakkoli, Kabeh Mohsenzadegan, Jean Chamberlain Chedjou, Kyandoghere Kyamakya

**Affiliations:** Institute for Smart Systems Technologies, University Klagenfurt, A9020 Klagenfurt, Austria; kabehmo@edu.aau.at (K.M.); Jean.Chedjou@aau.at (J.C.C.); kyandoghere.kyamakya@aau.at (K.K.)

**Keywords:** ODE Solver, OpenCL, Parareal, parallel/multi-core computing, sensing systems, heterogenous embedded systems

## Abstract

Solving ordinary differential equations (ODE) on heterogenous or multi-core/parallel embedded systems does significantly increase the operational capacity of many sensing systems in view of processing tasks such as self-calibration, model-based measurement and self-diagnostics. The main challenge is usually related to the complexity of the processing task at hand which costs/requires too much processing power, which may not be available, to ensure a real-time processing. Therefore, a distributed solving involving multiple cores or nodes is a good/precious option. Also, speeding-up the processing does also result in significant energy consumption or sensor nodes involved. There exist several methods for solving differential equations on single processors. But most of them are not suitable for an implementation on parallel (i.e., multi-core) systems due to the increasing communication related network delays between computing nodes, which become a main and serious bottleneck to solve such problems in a parallel computing context. Most of the problems faced relate to the very nature of differential equations. Normally, one should first complete calculations of a previous step in order to use it in the next/following step. Hereby, it appears also that increasing performance (e.g., through increasing step sizes) may possibly result in decreasing the accuracy of calculations on parallel/multi-core systems like GPUs. In this paper, we do create a new adaptive algorithm based on the Adams–Moulton and Parareal method (we call it PAMCL) and we do compare this novel method with other most relevant implementations/schemes such as the so-called DOPRI5, PAM, etc. Our algorithm (PAMCL) is showing very good performance (i.e., speed-up) while compared to related competing algorithms, while thereby ensuring a reasonable accuracy. For a better usage of computing units/resources, the OpenCL platform is selected and ODE solver algorithms are optimized to work on both GPUs and CPUs. This platform does ensure/enable a high flexibility in the use of heterogeneous computing resources and does result in a very efficient utilization of available resources when compared to other comparable/competing algorithm/schemes implementations.

## 1. Introduction

The history of using differential equations has traces in calculus from the old Newton’s times. Since then it has evolved so much, and it is extensively used in many different branches of science and engineering. The numerical solving of differential equations with initial conditions is a classic problem, which has emerged before the computer invention and has various different usages in physics [1], engineering [2], chemistry [3], economics [4], biology [5], and several other disciplines. Specifically in sensors, ODEs are involved in various processing endeavors such as to detect anomalies in machines related sensor data [6], or to model nonlinear sensors like time-variant inductors [7] or piezoelectrically actuated microcantilever sensors [8], and/or to study sensors’ behavior or to optimize sensors’ performance. ODEs also used to find sensor’s optimal location [9]. Quorum sensing (QS), which is based on bacterial communication, can also be modeled with differential equations. Furthers, ODEs can also be used in self-organizing networks, self-diagnostic and environmental monitoring systems [10]. Hence, finding new ways for solving ODEs in shorter time can help to save, besides processing related energy consumption, both money and time, while using cheaper devices for better performing applications.

Differential equations have many different forms. In this paper, we do focus on ordinary differential equations (ODEs).

Equation (1) is showing an example of this type of differential equations:

(1) $$\dot{y}\left(t\right)=f\left(t,y\left(t\right)\right);\;y\left(t_{0}\right)=\;y_{0},\;t\;\in \left[t_{0},t_{e}\right]$$

where y is a vector valued function of t (time), $y\left(t\right):\mathbb{R}\to {\mathbb{R}}^{n}$, *n* is dimension of problem, the time derivative of y, $\dot{y}\left(t\right)$, is a function of y and t, and the function f has the values domain $f:{\mathbb{R}}^{n}\to {\mathbb{R}}^{n}.$ Further, $y\left(t_{0}\right)=\;y_{0}$ is called the initial value. Thus, we do have a so-called initial value problem (IVP)) and $y_{0}$ is the starting point for calculations at $t_{0}$. The solving of Equation (1) shall calculate the values of y from $t_{0}$ until $t_{e}$.

We need to determine the problem’s solutions (i.e., y(t)) for all values of t within the interval [$t_{0}$,$\;t_{e}$], this thus starting from the initial value $y\left(t_{0}\right)$ up to the final value of the $y\left(t_{e}\right)$. The solution of Equation (1) can be found by applying various appropriate methods, which are either numerical or analytical. For those cases for which it is hard to find an analytical solution, one does usually then involve numerical methods. 

Regarding numerical methods, the simplest way to solve Equation (1) is to integrate the function *f* for over the study area (i.e., [*t_0_, t_e_*], provided the function *f* does satisfy the so-called Lipchitz conditions. A numerical solving can be implemented through a discretized version of Equation (1), which is given in Equation (2). In Equation (2), $y_{t+1}$ is the result of the calculation of one step. The calculation of one step is obtained by taking the previous value $y_{t}\;$ plus the integral of $f\left(t,y_{t}\right)$ from the previous time t up to the current time *t* + 1. For calculating the integral, various traditional methods like Euler, Runge-Kutta, etc. can be used:

(2) $$y_{t+1}=y_{t}+\;\int_{t}^{t+1}f\left(s,y_{s}\right)\cdot ds$$

where *s* is time between *t* and *t* + 1, and $y_{s}$ is value of function f in time s.

In the case of a single computing core, there is no problem to achieve an efficient usage of computing resources. In this one-core context, it is very easy and straight-forward to implement Equation (2) and, after the calculation of one step is finished, one does move on calculating the next step. The steps are solved in a serial manner and the result of each step is then be used for next steps’ calculations. However, when one works on a multi-core/parallel platform, that sequential model cannot be used anymore, as other available resources/cores/nodes would have nothing to do. 

There exists five different space-time parallel computing methods/schemes for implementing the difference equation Equation (2) [10]. Those five schemes are the following ones: domain decomposing, parallel solver, multiple shooting, direct time parallel, and multi grid.

In the domain decomposing scheme, one does separate, if possible, the problem into *n* sub-problems and solve each of them separately. This can be realized by integrating the ‘domain decomposition’ and the so-called waveform relaxation [11,12]. Basically, in this solver type, the problem domain is decomposed into overlapping sub-domains, and each domain is then solved separately [13]. Choosing the correct way for decomposing is very important for increasing the overall performance. Also, the ‘decomposing method’ can be varied due to the nature of Equation (1) [14,15].

A further approach is to use a parallel integration method. This is however not possible for single-step integration methods. See for example Equation (3), where the Euler method is presented. As one can see, for calculating $y_{t}$, one step is required, and this step cannot be separated (broken down) into smaller tasks/sub-steps for a separate implementation on different cores of a parallel system. The Δt is the calculation step. A lower value of Δt provides a higher accuracy for solving a given problem but it does however thereby increase the resulting calculation time.

(3) $$y_{t}=\Delta t\;\cdot \;\dot{y}\left(t\right)+y_{t-1}$$

Therefore, this above-named further approach, i.e., a “parallel integration method”, is only possible while using multi-steps methods such as Runge-Kutta or Adams-Bashforth integrators, which are classified as larger group of Generalized Linear Model (GLM) solvers. GLM solvers are explained in detail in Section 2.1.

Methods like Runge-Kutta are multi-steps iterated methods [16,17,18,19]; this means one can distribute calculations of each step on different computing nodes. But at the end of each step, the different computing nodes should send their results to one node to sum-up or combine them appropriately and then calculate a new value. This last part of the lastly described scheme does visibly create a bottleneck w.r.t. to the potential speeding-up of the solving of differential equations while using a multi-step method.

In the shooting methods which were introduced by Nievergelt in 1964 [20], Equation (2) is decomposed in the time direction into semi-linear boundary value problems. Smaller problems are then solved with higher accuracy in parallel, but the error will then be corrected in a serial operation. Although, this method is by definition sequential because of the integrated serial error correction. However, it normally does cost much less than the high-accuracy calculation of results on hole of integration area. Therefore, this brings real advantages in the perspective of solving any ODE problem [21].

Multigrid methods like the so-called “domain decomposition” can be used for solving non-linear ODE’s. The problem is discretized with finite approximations into sparse linear systems of equations. This linear system is later solved via stationary iterative schemes such as the Gauss-Seidel method [22,23].

In lastly described approach, one tries to solve the problem directly without any iteration. All iterations for solving *n* points will be put in one place in one matrix and the problem is then solved together at once [24]. 

Furthers, it can be observed that several scientific works have been undertaken in order to create new integration methods, which can provide ODE solvers with better possibilities for an efficient implementation on parallel platforms. These efforts mostly focused on creating the so-called Adams-Bashforth derivative methods such as parallel Adam-Bashford (PAB) and parallel Adam-Moulton (PAM) [25,26]. These last methods have shown very good scalability performance while increasing the number of computing nodes.

Today, most of modern computers have both n core CPUs (n-CPUs) and GPUs. The increasing power of CPUs and GPUs is mostly reached by increasing the number of computing nodes. Although the number of computing nodes has significantly increased in GPUs but also in n-CPUs, the need for algorithms capable to efficiently use the multi-core computing resources is strong. It has been shown that implementing “problem solvers” on parallel/multi-node platforms can speed-up the solving in many scientific fields such as fluid dynamics [27], finite elements methods [28], molecular dynamics research [29], applied physics [30], chemical kinetics [31], etc. 

On the other hand, for writing programs which can efficiently run on different computing architectures is not a trivial problem. For solving this concern, some middleware concepts/platforms which do support different types of n-CPUs or GPUs architectures have been developed and introduced. In this paper, we use the so-called OpenCL platform. It is possible, by using OpenCL, to run programs directly on CPU or GPU. However, this programming framework, like other similar frameworks has also its own restrictions. In this paper, we do introduce a new solver type/concept which does well fit and is integrated in the OpenCL platform. This novel ODE solver concept implemented through OpenCL has been extensively tested and benchmarked with other related competing famous/well-known algorithms from the most relevant literature.

This paper does present a very brief critical overview about related works in Section 2. Then, our novel ODE parallel-solver concept is introduced in Section 3. The implementation system architecture in OpenCL, which does support the running application of our novel solver concept is explained in Section 4. Then, extensive experiments and a comprehensive benchmarking are presented and discussed in Section 5 and Section 6. To finish, comprehensive concluding remarks, which summarize the quintessence of the results obtained, are presented in Section 7. 

## 2. Related Works

As briefly explained above, we should search for multi-stage methods, which do have the potential for solving each stage of the problem, possibly independently of each other [32]. For this study, the knowingly best-performing multi-step algorithms have been selected for analysis and possibly benchmarking too. One of those methods is derived from the Runge-Kutta family and we call it “Iterated Runge-Kutta”. And two further methods are derived the from the Adams–Bashforth family, which are respectively called “Parallel Adams–Bashforth (PAB)” and “Parallel Adams-Moulton (PAM)” [25].

### 2.1. General Linear Methods

The General Linear Method (GLM) as proposed by Butcher in 1966 was defined to generalize and integrate both Runge-Kutta (multi-stage) methods and linear multistep (multi-value) methods. During each step of the calculation, one considers r numbers of previous values and s stages. At the start of each step, we have input items from the previous steps as follows:

(4) $$y_{i}^{\left[n\right]},\;i=1,\;2,\;\ldots ,\;r$$

And during calculation of stages in one step, we have stage derivatives as follows:

(5) $$Y_{i},\;\;F_{i}\;,\;\;i=1,\;2,\;\ldots ,\;s$$

Thus, this method has the following variables for calculating the next stage n+1:

(6) $$y_{\left[n\right]}=\left[\begin{array}{c} y_{1}^{\left[n\right]}\\ y_{2}^{\left[n\right]}\\ \vdots \\ y_{r}^{\left[n\right]} \end{array}\right],\;\;y_{\left[n+1\right]}=\left[\begin{array}{c} y_{1}^{\left[n+1\right]}\\ y_{2}^{\left[n+1\right]}\\ \vdots \\ y_{r}^{\left[n+1\right]} \end{array}\right]\;,\;\;Y=\left[\begin{array}{c} Y_{1}\\ Y_{2}\\ \vdots \\ Y_{s}\; \end{array}\right],\;\;F=\left[\begin{array}{c} F_{1}\\ F_{2}\\ \vdots \\ F_{s}\; \end{array}\right]$$

These quantities are related to each other by the following equation, see Equation (7):

(7) $$\begin{array}{l} Y=h\left(A⊗I\right)\;F+\left(U⊗I\right)\;y_{\left[n\right]}\\ y_{\left[n+1\right]}=h\left(B⊗I\right)\;F+\left(V⊗I\right)y_{\left[n\right]}\\ F=f\left(Y\right) \end{array}$$

where ⊗ is tensor product, h is the step-size in $\left[t_{n},\;\;t_{n+1}\right]$, and A, U, B and V are constant matrices having the following respective dimensions:

(8) $$A\in {\mathbb{R}}^{s\times s},\;U\in {\mathbb{R}}^{s\times r},\;B\in {\mathbb{R}}^{r\times s},\;V\in {\mathbb{R}}^{r\times r}$$

In Equation (7), the result of the step ($y_{\left[n+1\right]}$) is calculated based on the previous values ($\;y_{\left[n\right]})$ and the stage values (F, Y). F is calculated directly from Y based on the Equation (1) definition. For those linear multistep methods for which previous values ($y_{\left[n\right]}$) are required, the starting vector can be calculated by one of the *n*th-order Runge-Kutta methods such as the so-called Dormand-Prince method (DOPRI) which do not need previous values. 

As Butcher explained [33], the customization of GLM is creating a different ODE solver, which can be customized to have properties of either the Runge-Kutta method by setting r=1 or the linear multistep method by setting s=1. For solving non-stiff ODE problems, some methods based on GLM have been created by customizing the parameters r and s and/or the matrices A, U, B and V.

For example, the classical fourth order of Runge-Kutta can be expressed in GLM with the following matrices:

(9) $$A=\;\left[\begin{array}{cccc} 0 & 0 & 0 & 0\\ \frac{1}{2} & 0 & 0 & 0\\ 0 & \frac{1}{2} & 0 & 0\\ 0 & 0 & 1 & 0 \end{array}\right],\;\;U=\left[\begin{array}{c} 1\\ 1\\ 1\\ 1 \end{array}\right],\;B=\left[\begin{array}{cccc} \frac{1}{6} & \frac{1}{3} & \frac{1}{3} & \frac{1}{6} \end{array}\right],\;V=\left[1\right]$$

And in the case of the second order Adam-Bashforth method, A, U, B and V can expressed as follows:

(10) $$A=\;\left[0\right],\;\;U=\left[\begin{array}{ccc} 1 & \frac{3}{2} & -\frac{1}{2} \end{array}\right],\;B=\left[\begin{array}{c} 0\\ 1\\ 0 \end{array}\right],\;V=\left[\begin{array}{ccc} 1 & \frac{3}{2} & -\frac{1}{2}\\ 0 & 0 & 0\\ 0 & 1 & 0 \end{array}\right]$$

By choosing a strictly diagonal or triangular matrix A, the stage calculation will be decoupled into s independent sub-systems. Therefore, in this case, the implementation of the solver on a parallel/multi-core system is much easier as the stage dependency is thereby significantly reduced. 

For example, if we have following A matrix:

(11) $$A=\;\left[\begin{array}{ccccccc} 0 & & & & & & \\ x & 0 & & & & & \\ x & 0 & 0 & & & & \\ x & 0 & 0 & 0 & & & \\ x & x & x & 0 & 0 & & \\ x & x & x & 0 & 0 & 0 & \\ x & x & x & 0 & 0 & 0 & 0 \end{array}\right]$$

(2,3,4) Stages and (5,6,7) Stages can be computed concurrently as those stage does not use value from each other, Therefore they can be solved in parallel way. This pattern can be seen in the parallel iterated Runge-Kutta (PIRK) or better in the step-independent methods like the PAB or the PAM [25] methods, where A=[0]. In this paper, we do also use the GLM solver to create our new solver by customizing the matrices A, U, B and V.

#### 2.1.1. Parallel Iterated Runge-Kutta

This method is defined according to [34,35,36] and is also based on the GLM method (Equation (7)). The matrices A and V have the following definition:

(12) $$A=\left[\begin{array}{cccc} 1 & 0 & \cdots & 0\\ \vdots & \vdots & \cdots & \vdots \\ 1 & 0 & \cdots & 0 \end{array}\right],\;V=\left[1\right]$$

The U matrix is calculated based on related Runge-Kutta method as explained in the previous sections. This method is a very precise method. But it is not using all resources when we have only one ODE equation. The number of steps can be changed during each iteration. Therefore, one can reach a significant speed-up while solving large problems needing too steps to calculate.

**Figure 1:**
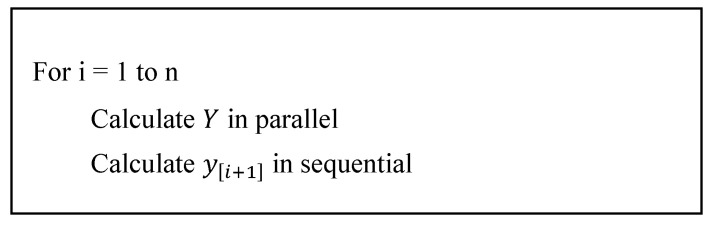
Implementation of the parallel Runge-Kutta algorithm. Stage values (Y) can be calculated in parallel but the step result needs to be calculated sequentially.

An implementation example of this model can be shown in Figure 1:

Figure 1 is showing the calculation flow of the different steps. The stage values (Y) can be done in a parallel way, but each processing unit needs to exchange information during the processing and at the end of each stage. Again, each node requires to exchange information with another specific node in order to sum up all steps and create the step value ($y_{\left[i+1\right]})$.

#### 2.1.2. Parallel Adams-Bashforth

This method was introduced by v.d. Houwen and Messina in 1998 [25]. Since then it has been further developed and optimized to be used in parallel platforms [37]. The Parallel Adams-Bashforth (PAB) is based on the Adams–Bashforth corrector by customizing the GLM with A, U, B and V matrices having the following values:

(13) $$A=\left[0\right],\;V=a\cdot b^{T},\;a=\left[\begin{array}{cccc} 1 & 1 & \ldots & 1 \end{array}\right],\;b=\left[\begin{array}{cccc} 0 & \ldots & 0 & 1 \end{array}\right]$$

**Figure 2:**
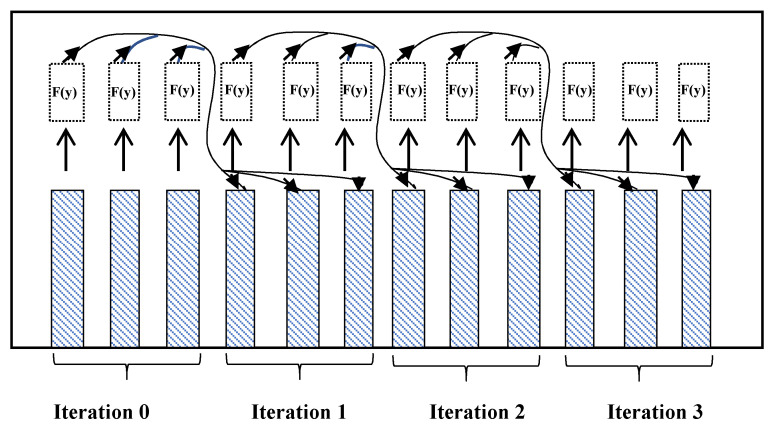
PAB execution and scheduling scheme on 3 processing units. The result value of each iteration is calculated and then the F value for the next iteration is computed. Those values will be propagated to the other processing units for the next iteration. In each iteration, 3 points of the problem are solved.

The U matrix is calculated based on the related Adams-Baschforth method explained above in the GLM section. It has been proved that by choosing those matrices in Equation (13), the PAB solver becomes super-convergent to the real solution of an ODE problem. Implementing this method on parallel system is not straight forward and requires a special scheduling. Figure 2 is showing a basic scheduling for running this method on 3 processing units. In each iteration, after find the results ($y_{\left[i\right]})$, the F values which are to use for the next iteration will be calculated. Thus, each iteration calculation can be done in a parallel way. But after finishing an iteration, each computing unit should exchange its information with other processors in its respective group of processors. This process will be continued until end of the calculation time (Figure 2). 

The PAB method can result in an improvement of the speed-up when compared to the Runge-Kutta method because, here, the communication between nodes can be done only at the beginning and at the end of running a stage. Therefore, it is very efficient to implement the PAB method on a parallel system. On the other hand, if we want to implement this method on an OpenCL platform, we do need a very good synchronization. This because the last node having the larger amount of calculations, the other nodes need to wait until it will finalize its calculations and only then let the other nodes synchronize themselves with latest values.

### 2.2. Multiple Shotting Methods

In this type of methods, as explained previously in the introduction section, the space-time domain is decomposed into smaller parts (sub-domains) and each subdomain is solved separately. The idea of creating this method is coming from Nievergelt in 1964 [20]. Since then, the method has been developed and extended by different researchers and it is mostly well-known as ‘Parareal’ algorithm [38,39,40].

The general implementation of this so-called “Parareal” algorithm is composed/constituted of two propagation operators:

1. The “Coarse approximation,” which is $G\left(t_{i},t_{i+1},y_{i}\right)$ with the initial conditions $y_{i}=y\left(t_{i}\right)$ with the step size $h_{g}$.
2. The “Fine approximation,” which is $F\left(t_{i},t_{i+1},y_{i}\right)$ with the initial conditions $y_{i}=y\left(t_{i}\right)$ with the step size $h_{f}$.

Where $h_{g}\gg h_{f}$, therefore the main difference between the above listed two propagation operators is their respective accuracy and the amount of time they do need to find the result as the coarse approximator has a larger step size.

The main algorithm consists of the following steps [12]:

1. Find the values of $y_{1},\;\ldots ,\;y_{n}$ by using $y_{i+1}=\;G\left(t_{i},t_{i+1},y_{i}\right)$ in a sequential way.
2. Copy the $y_{1},\;\ldots ,\;y_{n}$ values into $g_{1},\;\ldots ,\;g_{n}$ in parallel.
3. Find the $f_{1},\;\ldots ,\;f_{n}$ values by using $f_{i+1}=\;F\left(t_{i},t_{i+1},y_{i}\right)$ in parallel.
4. Update $y_{1},\;\ldots ,\;y_{n}$ in sequential with the following steps:
  - $gn_{i+1}=\;G\left(t_{i},t_{i+1},y_{i}\right)$.
  - $y_{i+1}=\;f_{i+1}+gn_{i+1}-g_{i}$.
5. Copy the $gn_{1},\;\ldots ,\;gn_{n}$ values into $g_{1},\;\ldots ,\;g_{n}$.
6. Go to the step 3 until you reach required precision.

This above-described algorithm is also sequential; for each iteration we do also need the values from the previous iteration. Thus, there is no real parallel-time integration as the sequential nature of the process is not removed. But the most expensive part is done in parallel (see Step 3) and solving that most expensive part in parallel will bring a significant advantage when increasing the number of computing nodes. One main disadvantage is, however, that this algorithm needs too many computing nodes to reach a good speed-up. Consequently, it is not efficient to implement it for a small number of computing nodes in the ranges like less than 8 or 16. 

### 2.3. Summary of the Main Previous/Traditional Methods

By comparing the properties of the above presented methods, there is one big difference amongst them. The GLM methods are optimized to be efficiently used in the context of parallel systems’ contexts having specified properties like “running schedule” and “number of processing units”. We implemented all these three above listed methods on an OpenCL framework in order to carefully analyze their respective performance along with related respective observed implementation restrictions. One restriction (weak point) is related to the poor scalability w.r.t. to the increasing number of cores. One does observe a very poor performance scaling of the algorithms while increasing the number of involved nodes for solving a problem. On the other hand, the Parareal method is showing a very good advantage when increasing number of cores; however, it is showing a very poor scaling performance in presence of a small number of cores (e.g. 8 cores or 16 cores). 

This observed gap has motivated us to create a new method based on both the Parallel Adam Bashforth method and the Parareal algorithm by using the advantages of both methods with a significantly higher compatibility with the OpenCL framework.

## 3. Our Novel Concept, the Parallel Adam-Moulton OpenCL

Our novel method, that we call the Parallel Adam-Moulton OpenCL method (we abbreviate it in PAMCL) is a modified version of the Adams-Bashforth method, which has a scheduling scheme like the PAB method (see Figure 2). This method is defined through the following equation for each group of computing units:

(14) $$A=\left[0\right],\;V=a\cdot b^{T},\;a=\left[\begin{array}{cccc} 1 & 2 & \ldots & g \end{array}\right],\;b=\left[\begin{array}{cccc} 0 & \ldots & 0 & 1 \end{array}\right]$$

where g is the group size of processors, and the number of previous values is g. Therefore, based on the number of processors in a group, the requirements to the previous values are different. The matrix U is calculated based on the related Adams-Baschforth method as explained in the GLM section. 

The starting vector for this algorithm is calculated by a suitable scheme like the DOPRI5 method [41], and each value of F  is approximated by using the previous steps of the solution vectors by using the Equation (7). For each calculation stage, we have a (h×g ) step size advantage w.r.t. to *h*. On the other hand, with a growing size of the “processing units group”, we need a higher order method for calculating the result values ($y_{\left[n+1\right]}$) by using an Adams-Bashforth algorithm. In this way, we do increase our accuracy without losing in performance. 

After calculating the result, we do need a corrector function based on the Adam-Moulton formula to correct the calculations of the previous steps. 

Based on both the predictor (see Adams-Bashforth) and the corrector (see Adam-Moulton), we can calculate the values of the local error truncation, which leads to the calculation of the optimal step-size for each of the g steps for the local group, and the global step-size can change by synchronizing the groups after *m* steps calculation, where m is typically larger than g.

A sample implementation of the explained algorithm based on Equations (7) and (14) can be described as follows:

1. Define the number of CPUs in group (g) based on current hardware restrictions.
2. Define both work group step size and work item step size.
3. Solve the starting points by using for example “DOPRI 5” and save them in $X_{1},X_{1},\;\ldots ,\;X_{g}$, and their corresponding derivations as $F_{1},F_{2},\;\ldots ,\;F_{g}$.
4. Update $X_{i+1},\;X_{i+2},\;\ldots ,\;X_{i+g}$ in parallel through the following steps:
  - Calculate the derivation F by using Equation (7), Equation (14) and save in $F_{i+1}$.
  - Wait for all values of $F_{i+1}$, …, $F_{i+g}$.
  - Update the $X_{i+1},\;X_{i+2},\;\ldots ,\;X_{i+g}$.
  - Calculated the error for each computing unit and then update the global step size.
  - Synchronize all computing units and update value in global variable.
5. Go to the step 4 until all values calculated.

In previous, our implementation will be divided into 4 different parts:

(1) Calculating the gradient values for the next estimation points.
(2) Transferring the gradient vectors into the global memory.
(3) Estimating the next solution vector through an adapted Adams-Bashforth algorithm base on Equation (14) weights.
(4) Calculate local truncation error to adjust the step-size.

After these above listed main 4 steps, all local groups will be in synch (i.e., synchronized) for starting the next step. As we can see, most of the complexity of this algorithm is related to the correct usage of local and global variables, and most of the calculations will be solved in steps 3 and 4 depending on the problem size and the number of groups members.

An important effort in each calculation is to make the steps completely independent and create tasks with the same size in order to decrease the synchronization time between work groups. 

Furthermore, by increasing the number of computing units to more than 32, we found out that this method then becomes inaccurate. For increasing the accuracy, the previous method is combined with an algorithm which is explained in Section 2.2, where the propagation factors G and F are replaced by a new suggested propagator factor. Therefore, the explained previous algorithm will run on local groups of processing units and grouped together does create a Parareal solver.

## 4. System Architecture

This computing system is designed based on the OpenCL platform. OpenCL is a heterogeneous computing platform, which is a framework for writing programs that are executed across heterogeneous platforms consisting of CPUs, GPUs, and other processors. 

OpenCL includes a language (based on C99) for writing kernels (functions that execute on OpenCL devices) and APIs that are used to define and then control the platforms. OpenCL supports parallel computing by using a data-based and task-based parallelism. OpenCL has been adopted by Intel, AMD, NVidia, and ARM. Academic researchers have investigated the possibility of automatically compiling OpenCL programs into application-specific processors running on field programmable gate arrays (FPGA) [42]. Also, commercial FPGA vendors are developing tools to translate OpenCL to run on their FPGA devices. This feature of OpenCL motivates us to use this platform for implementing ODE solvers. But it is not possible to use this platform directly for different programming languages and web applications. Therefore, for the sake of expandability of the system, one application cloud, as shown in Figure 4, has been designed. This cloud application is creating a computing platform/network for solving ODE problems across a network of computing units.

**Figure 3:**
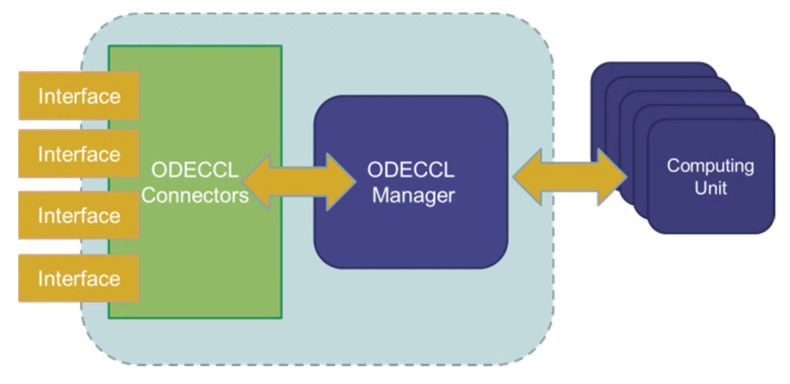
Solver system architecture. The interfaces provide the possibility of define specific tasks for the ODE solver. The ODE Computing platform OpenCL (we call it ODECCL) is composed of three parts. The manager will be assigning/allocating resources depending on their availability. Tasks are defined based on the different interfaces of ODECCL.

Figure 3 is showing our global system architecture. It is composed of 3 main components. The ODE Computing platform OpenCL (we call it ODECCL) connectors are responsible for connecting the manager to the interfaces and getting/collecting tasks originating from different applications and destined to ODECCL. 

After a task has been validated in the system, it will be sending message(s) to the ODECCL manager. The ODECCL Manager is responsible for managing, scheduling and supervising tasks. Each task is scheduled based on its respectively needed computing unit’s calculation power (Flops) and the communication cost. Computing units have the responsibility to execute tasks on the available OpenCL resources; therefore, it is possible to execute tasks both on CPU, n-CPU, and GPU at the same time (i.e., within the same parallel computing networked infrastructure). 

**Figure 4:**
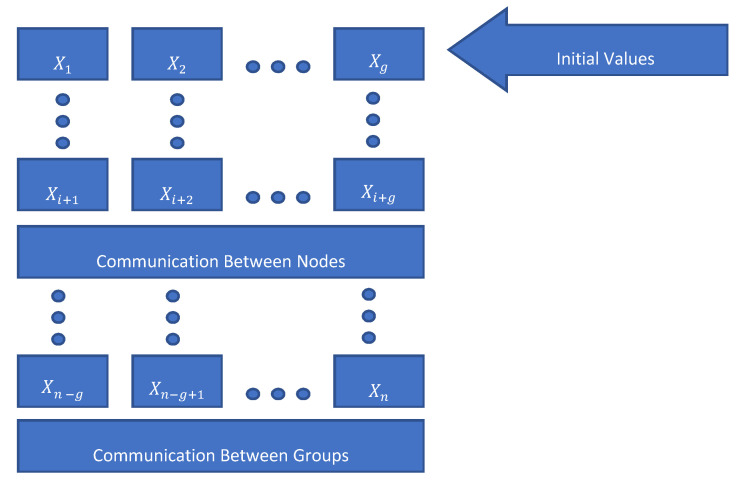
Scheduling scheme within/by the ODECCL system. Inside a group of processors, a stage will be processed, and between groups, steps will be calculated and synchronized.

Figure 4 does show the task scheduling concept in the ODECCL system. The scheduling is done between *m* groups and each group has *g* computing units (group units). After each stage, the computing units do exchange information in order to update their respective states and calculate the next gradient value.

From a technical perspective, ODECCL has been implemented using Visual Studio C++ and the OpenCL library provided by Nvidia has been included to the project. Each solver which is used in the experiments has been implemented as a kernel. 

For example, if the use of the overshooting algorithm is not required, our system does use kernels without the overshooting parts. The manager part of the application is responsible to load the correct kernels for each problem and it is also responsible to create the groups and assign the required processing units to the created groups. The manager is also providing facility (i.e., infrastructure) to retrieve data from the different interfaces and load/download them from/to the kernels.

## 5. Numerical Experiments

For testing our computing system described in Section 4, we select three different types of ODEs. These three equations are shown in Equations (15)–(17). This does correspond to respectively solving Rayleigh (see Equation (15)), Rössler (see Equation (16)), and JACB (see Equation (17)) dynamic models.

The Rössler function is sensitive to inputs; see Equation (16). The parameters of the Rössler function are selected to have a chaotic behavior. This shall test our system in presence of small changes in initial conditions; thereby we can show significant variation in the observed behavior. This is also good to show the accuracy of the algorithm(s) while considering different computing units. Furthers, Equations (16) and (17) are selected as stiff ODE problems to test the system stability and for comparing our results with those from other related scientific works. They are very sensible to errors and a small error will/can change their respective final result.

(15) $$\begin{array}{l} \frac{d^{2}x}{dt^{2}}-\varepsilon_{1}\left[1-({\frac{dx}{dt}}^{2}\right)]\left(\frac{dx}{dt}\right)+\omega x+k\sin\left(2\pi f_{1}\right)=0\\ \varepsilon_{1}=2.3\;,\;\omega =1\;,\;k=\;2.398\;,\;f_{1}=0.004\;\\ X_{0}=\left[-0.5,0.1\right]\;,\;t\in \left[0,20s\right] \end{array}$$

(16) $$\begin{array}{l} \frac{dx}{dt}=\;-y-z\\ \frac{dy}{dt}=x+ay\\ \frac{dz}{dt}=b+z\;\left(\;x-c\right)\\ a=0.2,\;b=0.2,\;c=5.7\\ X_{0}=\left[1,1,0\right],\;t\in \left[0,20s\right] \end{array}$$

(17) $$\begin{array}{l} \frac{dx}{dt}=\;y\cdot z\;\\ \frac{dx}{dt}=-x\cdot z\;\\ \frac{dz}{dt}=-0.51\;x.y\\ X_{0}=\left[0,1,1\right],\;t\in \left[0,7.5s\right] \end{array}$$

All models have been implemented on the following platform: Windows 10 PC with Intel Core i7 7700K as CPU, double Nvidia GeForce GTX 1080 TI with 8GB RAM as GPU and 64GB RAM. The Intel Core i7 7700K has 4 cores or 8 logical threads. The Nvidia GeForce GTX 1080 TI has 3584 cores which can be used for parallel computing. 

Table 1 does show the respective kernel configuration for each of the solvers considered. As one can see, our model (PAMCL) does integrate two different solvers. The first solver has no overshooting algorithm and the second one has this ability of overshooting to fill up the problem of using a large number of cores. Regarding the first solver, the workgroup size is the same like the one of PIRK and PAM, and the number of working items is dependent on the number of available cores. But in the second solver of PAMCL the number of workgroups is variable and we always keep the number of internal working items of each workgroup to be 8, as this number is the most efficient solver w.r.t to the number of cores (as this is illustrated in Table 2).

The computing time results of testing of our novel algorithm can be seen in Table 2, where they are provided for different differential equations to be solved. One can see by adding more cores results in increasing the performance of the system. But after 16 cores the performance increase is no more exponential, on one hand, and the overhead of the algorithm does significantly increase. Indeed, the decrease in computing time performance consecutive to increasing the number of cores 8 times, namely changing it from 8 cores and 64 cores, is just of 3 times, although one has added 8 time more cores. This poor gain in the resulting performance through adding more cores is even worser in when the number of cores is much higher. 

## 6. Comparison of the Novel Concept (PAMCL) with Previous/Related Methods 

**Figure 5:**
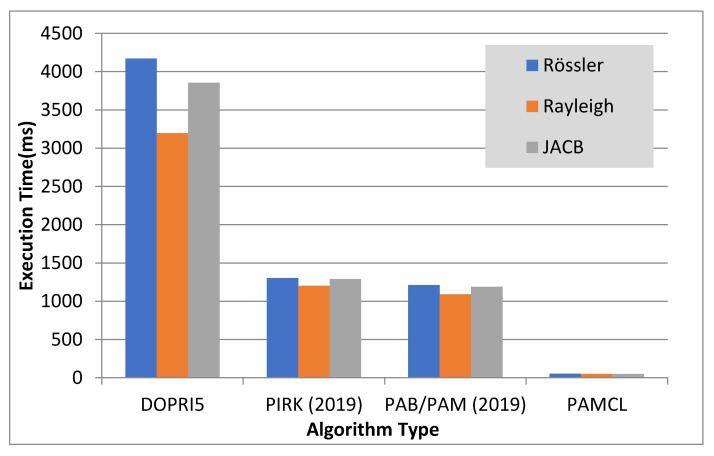
Comparison of the execution times of different problems while using different algorithms. The maximum computation error to stop the computing process is 0.001. All algorithms are executed on GPU for solving the Rössler equation. DOPRI5, PIRK, PAB/PAM and PAMCL are using 1, 8, 16 and 500 GPUs.

As explained in the previous sections, we define the speed-up by considering equal tasks on different cores. Therefore, our speed-up concept is not directly comparable to that of DOPRI or that of other algorithms, because most of them are rather running on a single-core computing unit. But for the sake of a better understanding, we calculate an “equivalent speed-up” performance w.r.t. one single computing unit. Figure 5 has been generated accordingly. As we can see, the PAMCL algorithm (i.e., our novel concept) can provide a much better speed-up depending on available free cores, either GPU or n-CPU. This speed-up, for 500 GPU cores, can reach up to 60x faster than the normal DOPRI5 algorithm, which is used in commercial ODE solvers like Matlab on the same computer/CPU.

**Figure 6:**
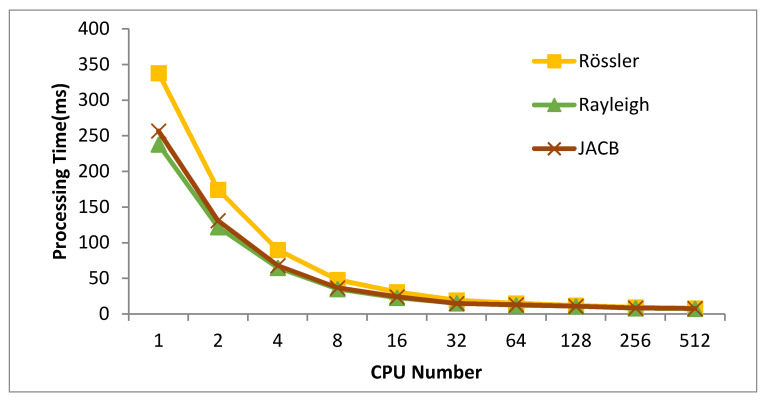
Effect of the number of processing units on the computing performance (for our novel approach PAMCL) for solving the Rössler attractor in GPU. The maximum error is 0.01.

In Figure 6, for our novel method PAMCL, the evolution of the processing time w.r.t. the CPU number is shown. As could be expected, according to the Gustafson’s law [43], the system performance is not increasing linearly and the speed-up does reach a saturation after 16 computing units. 

Further, in case of more complex equations, the advantage of our novel algorithm will increase, because the ratio between processing time and communication time to other processing units is higher.

While comparing CPU and GPU performance for solving differential equations, we do further observe that an implementation of the DOPRI algorithm on CPU is much faster than on GPU. However, while using the PAMCL algorithm, it does provide again more advantages w.r.t. a normal execution of the DOPRI algorithm on CPU (see Table 3).

For each model which has been explained previously, one has created a respective own kernel. The main problem regarding Runge-Kutta and PAM is that both methods have restrictions related to the number of cores as the number of cores increases beyond 8, both lastly named methods become worse w.r.t. reaching the required accuracy. Therefore, in order to reach the required error level, they will need more (i.e., additional) iterations, which does result logically in more computing time.

As we can see in Table 4, the increasing performance which are demonstrated in previous table is due to the fact of using more global and local memory. Indeed, our model used respectively more memories compare to other methods. 

**Figure 7:**
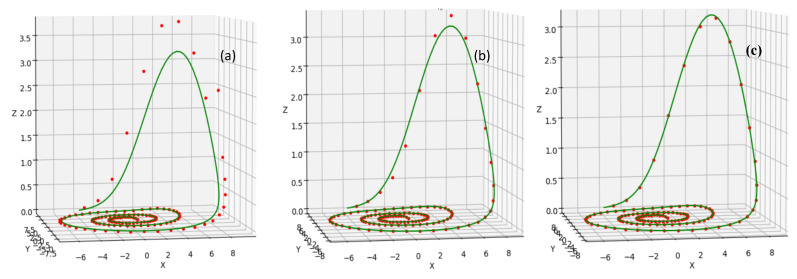
Showing the effect of iterations on the convergence towards the solution of the Rössler Equation (17). The green line is showing the expected solution, and the red dots are showing the PAMCL estimation at different iteration steps. For all 3 sub-figures (i.e., from left to right), the same parameter settings were used but 25 points are used in Sub-Figure (**a**), 50 points are used in Sub-Figure (**b**), and 100 points are used in Sub-Figure (**c**). Those points are calculated in a parallel way. It is visible that the estimation of model is changing from the expected results and an increasing number of does increase the model accuracy, but does also increase the calculation time.

As explained previously, for extending the PAMCL to a higher number of cores, we use the Parareal algorithm. This algorithm has its own drawback, as one needs to define the required iteration numbers needed to reach a convergence to the correct answer (see Figure 7). Thus, increasing the speed-up of PAMCL by using/integrating the Parareal algorithm will also have its own similar drawback.

## 7. Possible Extension of the PAMCL Model for also Solving PDE’s

The suggested model (PAMCL) can also be used for solving PDE models. The main difference in solving PDE’s lies essentially in the fact that in a PDE one has more dimensions. Therefore, it is possible to use/involve concepts such as Domain Decomposition, Waveform Relaxation [44] or multiple shooting method [45,46], which are used for solving either ODEs and PDE problems. 

Regarding the Domain Decomposition approach, we can separate our domain into sub-domains. Then, in this case, we can solve each subdomain separately and combine the results of each domain to get the final solution.

As we have seen in previous sections, our model is not compatible with such a way of solving the problem and we use the multiple-shooting method for solving the ODE. Therefore, the best way to solve PDE problems in our model is to keep the domain and create subdomains in time (Multiple shooting method). This process can be expressed into the following steps:

1. Converting PDE problem into ODE problem. Customize solver to solve PDE in parallel on n-CPUs/GPUs groups.
2. Define the step-size of both coarse and fine estimators to find the solution of PDE between groups.
3. Solving the PDE sequentially using the coarse estimator in the overall time span.
4. Solving the PDE in parallel using fine estimator in each of the split time spans.
5. Update the values in each of the split time spans by using the coarse estimator sequentially.
6. Go to step 3 until we reach the required precision.

In step 1, we need to approximate/transform the PDE problem into an ODE problem. The approximated ODE problem now can be solved on our platform. This step normally requires setup parameters, calculates boundary conditions, and solves matrix solutions. Therefore, we need custom the solver for PDE solving. Each step of the PDE will be processed on group of CPUs\GPUs. In step 2, we a have similar implementation of PAMCL, we used both fine and coarse estimators to reach the final solution. First results are approximated, and later, by using the fine estimator they will be corrected. This process can be continued until the overall model reaches the required precision.

## 8. Conclusions

Using our novel PAMCL method for solving ODEs on an OpenCL framework does increase the performance. Compared to other solvers, our novel algorithm (PAMCL) is displaying very good behavior and does converge always to the exact solution. By choosing the correct interpolations and adjusting the weights, the algorithm can perform much faster calculations with the required precision. But still, the communication between computing units requires more optimization, and more unused resource do still exist in the system.

Solving these problems can provide much better performance w.r.t to the current status of the system. Also, as we see in the implementation part, defining equal tasks (by definition within the PAMCL algorithm) can increase the overall performance by decreasing task scheduling amongst nodes and does also increase the performance on a GPU like architecture, as an execution of branches on such structures are costly. 

Also, by increasing the number of multi-stages, the calculation becomes more complex and it requires more resource to calculate values. This phenomenon occurs in all multi-step algorithms and it is required to provide load balancing between local workgroups and global workgroups.

## Figures and Tables

**Table 1:** The kernel configuration for each of the solvers. * The PAMCL solver has two different types of kernels, the first one without overshooting and the second one with overshooting. If the number of cores is more than 32, the second kernel type is the one to be used.

Solver/Parameter	Work- Groups	Work Item	Kernel Type Number
PIRK	1	Depends on available cores	1
PAM	1	Depends on available cores	1
PAMCL	Variable	Depends on available cores divided by the number of Work-Groups	2 *

**Table 2:** The execution time of PAMCL for different selected differential equations. The increase in number of cores does result in a decrease of the respective processing time. But by increasing the number of cores, this does result in more communication overhead amongst the cores.

Number of Cores on GPU	RAYLEIGH (ms)	JACB (ms)	RöSSLER (ms)
1	238.0	257.1	338
2	122.0	131.1	174.46
8	35.0	36.7	48.1
64	12.4	13.1	15.2
512	7.4	7.8	8.5

**Table 3:** Comparison of the “average computation times” on CPU and GPU while using different solver algorithms for solving the Rössler equation.

Method/ Algorithm	Error 0.01		Error 0.001		Error 0.0001	
	Time (ms) on CPU	Speedup (i.e., on GPU)	Time (ms) on CPU	Speedup (i.e., on GPU)	Time (ms) on CPU	Speedup (i.e., on GPU)
Dopri5	593.11	1x	4171.01	1x	41860.01	1x
PIRK (2019)	169.23	3.50x	1301.02	3.22x	15013.89	2.79
PAM/PAB (2019)	129.12	4.26x	1210.73	3.41x	11540.44	3.62
PAMCL	6.93	69.43x	51.83	80.47x	439.82	95.17x

**Table 4:** Comparison of “memory usage” w.r.t the target error while involving different solver algorithms for solving the Rössler equation.

Method/ Algorithm	Error 0.01		Error 0.001		Error 0.0001	
	Global Memory	Local Memory	Global Memory	Local Memory	Global Memory	Local Memory
Dopri5	18 KB	500 B	200 KB	500 B	2.1 MB	500 B
PIRK (2019)	19 KB	1.5 KB	210 KB	1.5 KB	2.2 MB	1.5 KB
PAM/PAB (2019)	22 KB	2.5 KB	250 KB	2.5 KB	2.6 MB	2.3 KB
PAMCL	32 KB	4 KB	350 KB	4 KB	4.8 MB	4 KB
